# Unapparent systemic effects of regional anticoagulation with citrate in continuous renal replacement therapy: a narrative review

**DOI:** 10.1186/s13613-023-01113-0

**Published:** 2023-03-11

**Authors:** Willem Boer, Walter Verbrugghe, Eric Hoste, Rita Jacobs, Philippe G. Jorens

**Affiliations:** 1 Department of Anaesthesiology, Intensive Care Medicine, Emergency Medicine & Pain Medicine, Ziekenhuis Oost Limburg ZOL, Genk, Belgium; 2 Department of Critical Care Medicine, Antwerp University Hospital, University of Antwerp, Edegem, Belgium; 3 Intensive Care Unit, Department of Internal Medicine and Paediatrics, Ghent University Hospital, Ghent, and Research Foundation Flanders (FWO), Ghent University, Brussels, Belgium; 4 Department of Critical Care Medicine, Antwerp University Hospital, LEMP, University of Antwerp, Edegem, Belgium

**Keywords:** Citrate anticoagulation, Calcium balance, Hormones, Phosphate, Magnesium, Review

## Abstract

The use of citrate, through reversible binding of calcium, has become the preferred choice for anticoagulation in continuous renal replacement therapy in the critically ill patient. Though generally considered as very efficacious in acute kidney injury, this type of anticoagulation can cause acid–base disorders as well as citrate accumulation and overload, phenomena which have been well described. The purpose of this narrative review is to provide an overview of some other, non-anticoagulation effects of citrate chelation during its use as anticoagulant. We highlight the effects seen on the calcium balance and hormonal status, phosphate and magnesium balance, as well as oxidative stress resulting from these unapparent effects. As most of these data on these non-anticoagulation effects have been obtained in small observational studies, new and larger studies documenting both short- and long-term effects should be undertaken. Subsequent future guidelines for citrate-based continuous renal replacement therapy should take not only the metabolic but also these unapparent effects into account.

## Background

Citrate anticoagulation has become the preferred choice for anticoagulation in continuous renal replacement therapy (CRRT) [1]. Despite occasional evidence to the contrary [2] it seems to reduce bleeding and prolongs the circuit lifespan compared to heparin [3]. Since its use has become widespread, several excellent reviews have been published describing the basics of regional citrate anticoagulation, concomitant acid–base disorders and the effects and management of citrate accumulation and overload [4, 5]. It stands to reason that calcium chelation not only has effects on the clotting cascade but also on many other processes mediated by fluctuations in ionized calcium (iCa). The delicate interplay between iCa, parathyroid hormone (PTH) and bone metabolism [6], for example, is undoubtedly subject to changes in the former caused by citrate chelation. Even a minor decrease in systemic iCa can trigger PTH release, mobilizing calcium (Ca) from skeletal stores within minutes [6, 7]. Considering how widely and for how long citrate anticoagulation has been practiced, evidence describing these effects in citrate anticoagulation in CRRT is surprisingly sparse. Similarly, effects on calcium, magnesium, and phosphate balance have all been described, though evidence here too is limited. Furthermore, calcium chelation has been documented to have effects, mediated through reducing iCa, pointing to an attenuation of inflammation and limiting systemic oxidative stress [5]

The purpose of this narrative review is to provide an overview of these other systemic effects of regional citrate anticoagulation. Firstly, effects on calcium balance and hormonal status, specifically on PTH and vitamin D metabolism, are described. This is followed by a description of effects on magnesium and phosphate balance. The ultimate part of this review is devoted to the impact of citrate anticoagulation on inflammation and oxidative stress. In general, this review is limited to the effects of citrate in the context of acute kidney injury (AKI) patients and does not describe evidence in chronic hemodialysis. Although it is our experience that many centers in Belgium and the Netherlands use the CVVH modality, the goal of this review is to encompass both continuous convective and diffusive modalities.

## Regional citrate anticoagulation (RCA)

Citrate induces anticoagulation by reversible binding (chelation) of calcium, causing hypocalcemia in the extracorporeal circuit. As calcium is a necessary cofactor in both the intrinsic and the extrinsic pathways, as well as in the common pathway, clotting is prevented.

Citrate used for anticoagulation during CRRT does not affect thrombin generation, D-dimer, or platelet function and hence does not protect against heparin-induced thrombocytopenia [8]. Before re-entrance of circuit blood to the patient, the calcium concentration is restored by administering a calcium solution. Regional anticoagulation is the result. To ensure a clinically significant anticoagulant effect, it is generally accepted that iCa levels in extracorporeal circuit blood need to be below 0.35 mmol/L [9]. It is important to note that discrepant postfilter iCa values were found when using different blood gas analyzers [10, 11]. In clinical practice, measurements of iCa in postfilter samples may give misleading information, particularly as blood gas analyzers are not validated for these measurements in low ranges [10, 11]. This phenomenon may also have distorted the results of some studies, making comparisons speculative.

After citrate administration, calcium citrate complexes are formed. Clearance in diffusive modes increases with the dialysate flow, in convective modes with filtration flow [12]. Complexes that are not removed through the hemofilter return to the patient. In convective modes up to 60% of the citrate given prefilter is cleared via the hemofilter into the effluent (sometimes more in diffusive modes), the rest returning to the patient for metabolization [12]. Decreasing blood flow limits the amount of delivered citrate to the extracorporeal circuit. Limiting blood flows in convective modes in combination with high filtration rates may lead to a high filtration fraction. Therefore, higher blood flows resulting in a higher delivered citrate dose may be necessary in convective modes, compared to diffusive modes, to achieve similar levels of clearance [4]. The Kidney Disease Improving Global Outcomes guidelines recommend targeting an effluent flow of 20–25 ml/kg/h [1]. Before the blood from the circuit is returned to the patient, calcium is added to normalize iCa and coagulation. Numerous protocols for regional citrate anticoagulation are available, utilizing different citrate solutions and CRRT modalities (continuous veno-venous hemofiltration (CVVH), continuous veno-venous hemodialysis (CVVHD), continuous veno-venous hemodiafiltration (CVVHDF)[4].

## Acute kidney injury and altered mineral metabolism

AKI causes a number of changes in mineral metabolism and is associated with hypocalcemia, hyperparathyroidism, hyperphosphatemia, and decreased 1,25–dihydroxy vitamin D (1,25D) [13].

Hypocalcemia in AKI is caused by decreased renal synthesis of 1,25D, resulting in decreased calcium gut absorption, decreased calcium reabsorption in the kidneys, and decreased release of calcium from bone [13–15]. Hyperphosphatemia may decrease serum calcium levels by sequestration of calcium, a phenomenon seen in case of massive tissue breakdown, accompanied by the release of large amounts of intracellular phosphate [13]. PTH production is increased in AKI due to hypocalcemia and low circulating 1,25D [13, 16], stimulating the secretion of PTH [17, 18], which both, when in normal range, cause negative feedback on the parathyroid glands. Pro-inflammatory cytokines cause upregulation of the calcium-sensing receptor in kidneys and parathyroid glands, changing the set point for calcium–PTH feedback regulation [19]. Hyperphosphatemia in AKI is caused mainly by decreased renal excretion of phosphate in most patients [13]. In bone, PTH, binding to receptors on osteoblastic cells, stimulates osteoclast activity, thereby inducing calcium release into the circulation [13]. This effect depends on the length of exposure to PTH, with chronic exposure leading to increased osteoclast activity and bone resorption, and pulsatile exposure leading to increased bone formation [13].

**Fig. 1:**
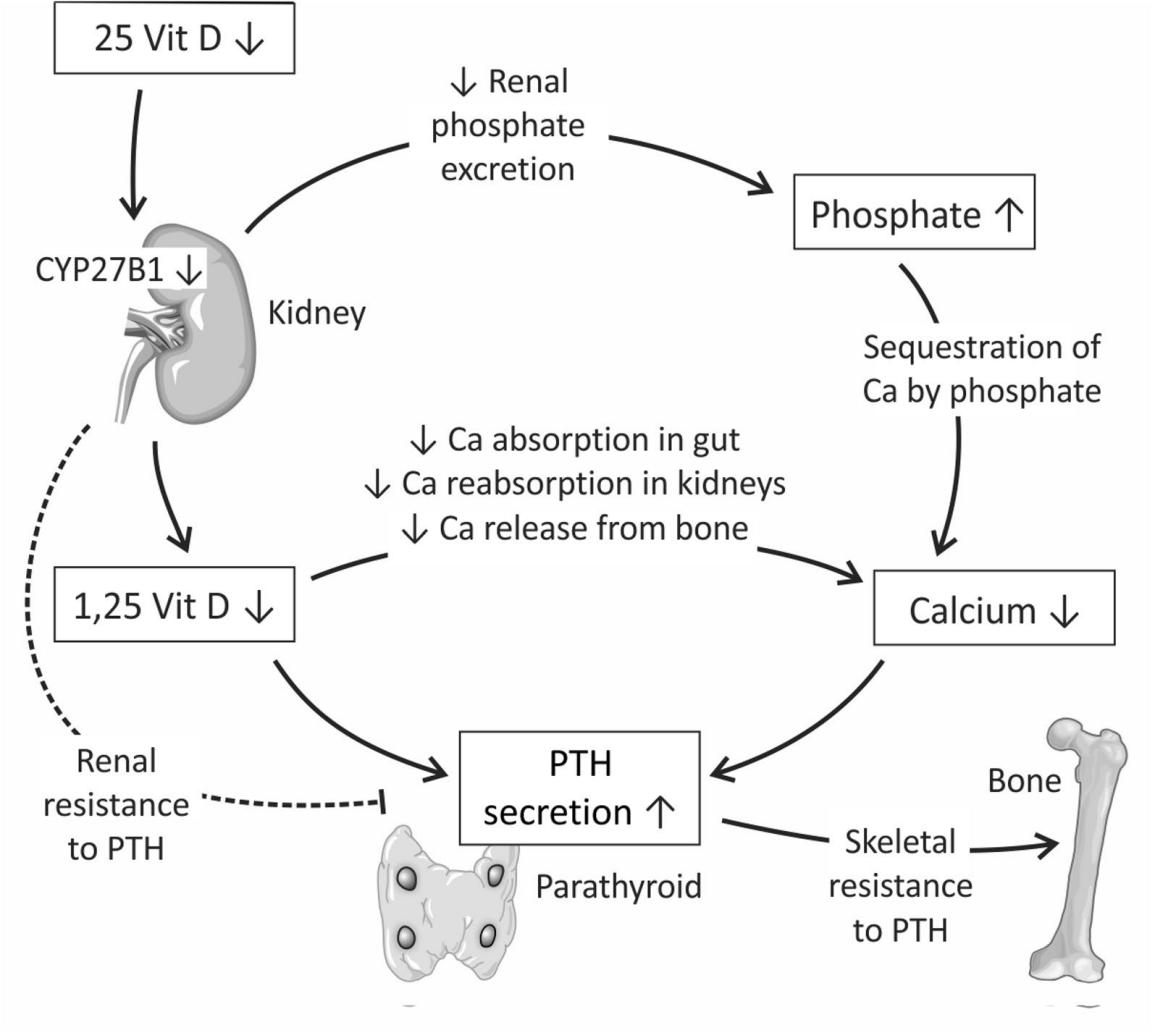
A simplified overview of changes in mineral metabolism in AKI. 1,25(OH)VITD3 deficiency in renal insufficiency is partly a consequence of 25(OH)VITD deficiency and a decrease in 1α-hydroxylase (CYP27B1) activity. This further leads to hypocalcemia, hyperphosphatemia, and hyperparathyroidism. Adapted from Leaf et al. [13]

**Fig. 2:**
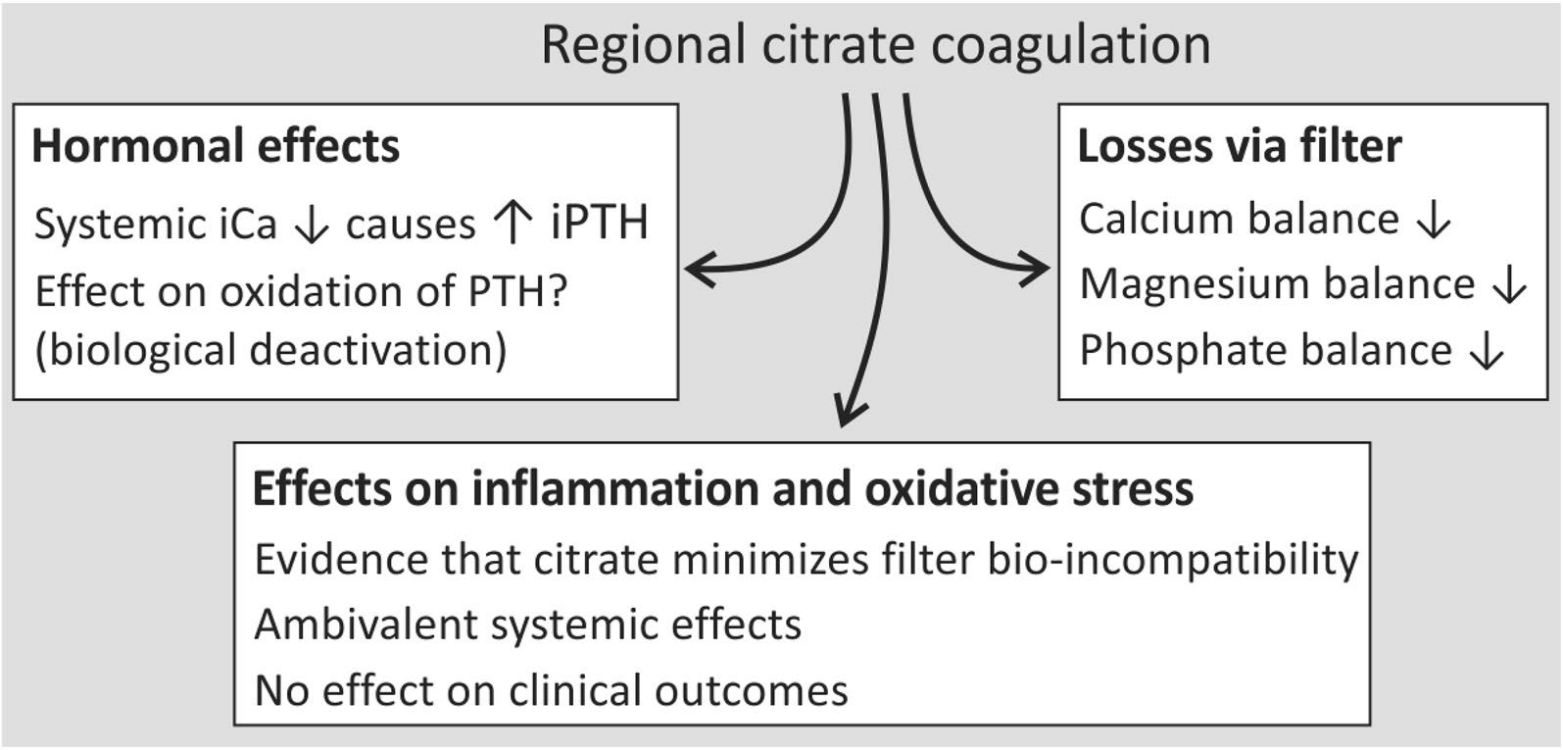
Unapparent systemic effects of RCA in CRRT

Although PTH levels are increased in patients with AKI, PTH often is unable to normalize circulating calcium levels, due to skeletal resistance to PTH [13, 16]. Similarly, there may be renal resistance to PTH in AKI, as demonstrated by low circulating 1,25D levels despite increased PTH, which in normal circumstances has positive feedback on 1,25D synthesis (Figs. 1, 2) [20]. However, the clinical significance of acutely increased PTH levels in AKI remains unclear [13]. A simplified overview of changes in mineral metabolism in AKI is provided in Fig. 1.

1,25D is derived primarily from hydroxylation of 25-hydroxy vitamin D (25D) in the proximal tubular cells of the kidney catalyzed by a cytochrome P450 enzyme, CYP27B1 [21, 22]. Decreased circulating 1,25D levels in patients with AKI could reflect either decreased substrate delivery of 25D to the proximal tubular cells and/or decreased CYP27B1 expression due to tubular injury [13]. The former could be the consequence of decreased circulating vitamin D binding protein (DBP) [23, 24], impaired enteral absorption of vitamin D in the setting of acute illness, or enhanced catabolism of 25D by the cytochrome P450 enzyme, CYP24A1 [13]. The clinical significance of decreased 25D and 1,25D levels both for predicting outcome of AKI and other clinical outcomes remains unclear [13].

## Citrate and calcium balance in CRRT

In citrate-based CRRT, insufficient replacement of calcium, lost as Ca-citrate complexes in the effluent, may lead to a negative Ca balance and systemic hypocalcemia [25–28].

Van der Voort et al. compared the effects of nadroparin-based and citrate-based CVVH (targeting citrate at 2.92 mmol/l and systemic iCa at 0.8–1.0 mmol/l), in critically ill patients with AKI, finding early initial iCa differences between the 2 groups, with significantly lower values in the citrate group [25]. These values equalized within 48 h, but the citrate-based group had a statistically significant negative daily calcium balance compared to the nadroparin group [25]. Brain et al. reported similar findings for the calcium balance in a CVVHDF model comparing heparin anticoagulation to citrate-based anticoagulation, here with a mean citrate dose of 2.42 mmol/l of blood and systemic iCa targeted at 0.8–1.1 mmol/l [26]. The mean calcium loss was 4.01 mmol/h from citrate-anticoagulated circuits versus a gain of 0.24 mmol/l from heparinized circuits. Despite the instigated calcium replacement regimen, citrate patients suffered a mean loss of 1.12 mmol/h [26]. Zheng et al. modeled the calcium requirements in citrate CVVH in a study consisting of two parts, first defining the determinants of calcium supplementation after which a two-phase calcium supplementation protocol was proposed and validated in critically ill patients [27]. Loss of calcium in the extracorporeal circuit and the increase in citrate-calcium complexes in vivo were the main determinants of calcium supplementation. With the implementation of a two-phase mathematical model, systemic calcium levels were maintained within the normal range [27]. Boer et al. compared the effects of low-dose (2.5 mmol/L) vs. high-dose (4.5 mmol/L) citrate for 24 h, targeting postfilter ionized calcium (pfiCa) of 0.325–0.4 mmol/L vs. 0.2–0.275 mmol/L over the first 24 h of CVVH [28]. The systemic dose was targeted at iCa > 1.0 mmol/L and calcium compensation was set at 100% on the hemofiltration device. The 24 h Ca balance was negative in the high citrate group but not in the low citrate group, when only considering the calcium compensation (set at 100%) provided by the dialyzer machine [28]. After taking the extra, physician prescribed, Ca supplementation into account, the Ca balance was positive in both groups, more so in the high-dose group. In the high-dose group 70% of patients received Ca supplementation vs. 31% in the low group. Calcium loss via effluent was found to be a citrate dose-dependent phenomenon. Calcium compensation on the dialyzer machine fell short at higher doses and extra physician-ordered calcium supplementation then proved indispensable [28].

In extreme circumstances, for example, in prolonged ICU care with consequential long-term immobilization combined with long-term regional citrate anticoagulation for CRRT and thus prolonged chelation of calcium, hypercalcemia caused by bone resorption and immobilization can be masked [29–31]. This may result in bone loss, despite normal serum calcium levels [30, 31].

In summary, calcium balance is determined by a number of factors: loss via the circuit (which in turn is determined by citrate dose), effluent dose (in dialysis modes defined by dialysate flow, in convective modes by filtration flow), and calcium substitution strategy. Other factors are bone resorption in patients with long-term immobilization, calcium loss via feces, and residual diuresis as well as enteral and parenteral calcium intake. It should be noted that all studies described above only describe short-term effects, to a maximum of 48 h. None take calcium excretion via feces into account and only one, a non-randomized study, includes calcium loss via residual diuresis [25]. Based on the available evidence, to minimize the risk of a negative calcium balance, it seems that systemic iCa during citrate CRRT should be targeted above 1 mmol/L though there is no evidence for targeting supranormal values. Similarly, to minimize net citrate dose, settings should be applied utilizing the lowest feasible blood flows. Although there are no data on calcium balance in CVVHD, theoretically this modality has the advantage that it requires lower blood flows than convective modalities. It should be noted that effects on calcium balance are not necessarily accompanied by clinical hypo- or hypercalcemia. Long-term randomized studies describing the effects on calcium balance are absent.

## Hormonal effects of citrate

The negative Ca balance and systemic hypocalcemia in citrate CVVH can lead to further activation of parathormone (PTH) which may already have been activated due to renal dysfunction [25, 28, 32]. Van der Voort found that median systemic iPTH was significantly higher in the citrate group (iCa at 0.8–1.0 mmol/l) compared to both the nadroparin group and the control group without renal failure [25]**.** Raimundo et al. maintained systemic iCa between 1.12 and 1.20 mmol/l in 30 consecutive critically ill patients treated with citrate-based CRRT and found that iPTH levels remained stable [32]. A mixed-effects model demonstrated that each 0.1 mmol/l increase in serum iCa concentration was associated with a 31.2% decrease in iPTH [32]. Boer et al., when comparing the Ca and Mg balance in high and low citrate doses, found that iPTH was increased, both at the baseline and at 24 h [28]. In both groups PTH declined during the first 24 h of CVVH [28]. Baseline iPTH was lower than the 264 pg/ ml reported in the Van der Voort study, but higher than the 67 pg/ml in the study by Raimundo. The differences in PTH values, particularly the lower values in the Raimundo study, could reflect differences in serum iCa values, which were consistently higher in the Raimundo study, although between-assay differences must be considered.

Critical illness-related oxidative stress leads to the oxidation of PTH. While the non-oxidized form of PTH (noxPTH) is a full agonist of the receptor, oxidized PTH (oxPTH) loses its PTH receptor-stimulating properties [33]. The standard iPTH measurements do not discriminate between bioactive noxPTH and oxPTH. In the Boer study, biologically active noxPTH did not change significantly over time [28]. However, the pattern of oxPTH was similar to that of iPTH, suggesting that the decline in iPTH may be attributed to a decline in biologically non-active oxPTH, e.g., to a decline in oxidative stress [28]. Although knowledge of the metabolism of oxPTH in the clinical setting is limited and most studies took place over longer periods, for example, in chronic hemodialysis (HD) populations, worse outcomes were found in patients with higher oxPTH values, possibly reflecting the effects of higher oxidative stress [34]. Citrate may limit oxidation of noxPTH to oxPTH [5, 35] though other factors may be at play. (Table 1 provides an overview of studies describing the effects on calcium balance and iPTH in RCA CRRT.

**Table 1:** Studies describing the effects on calcium balance and iPTH in RCA CRRT

Author	Van der Voort (25)	Brain (26)	Zheng (27)	Boer (28)	Raimundo (32)
Modality	CVVH	CVVHDF	CVVH	CVVH	CVVHD
Follow-up (hours)	48	24	24	24	48
Numbers included	9 patients in nadroparin vs 11 in citrate	26 heparin, 22 citrate circuits in 13 patients	20 patients in discovery cohort, 97 in validation cohort	17 high-dose, 18 low-dose citrate	30 patients
Comparison	Nadroparin vs citrate	Citrate vs heparin	Two-phase citrate model	High-dose vs low-dose citrate	Citrate only
Citrate dose (mmol/L)	2.92	2.42	4.0	4.88 vs 3.08	4.0
Systemic iCa (mmol/L)	0.8–1.0	0.8–1.1	1.0–1.2	> 1.0	1.12–1.2
Ca balance	Negative compared to nadroparin	− 4.01 mmol/h vs + 0.24/h	− 6.1 mmol/h	− 0.41 vs -0.05 mmol/h	–
Ca balance in citrate group including extra Ca	–	− 1.12 mmol/h	> 0	+ 0.69 vs + 0.19 mmol/h	–
iPTH (pg/ml)					
Baseline	28	–	–	222	66.5
24 h	42.3	–	–	162	88.3
48 h	30.0	–	–	–	85
Remarks	Calcium balance negative both with/without enteral feeding; non- randomized study	No enteral feeding Ca intake or loss via residual diuresis calculated	Study defining two-phase calcium compensation model when initiating RCA, only circuit and parenteral intake	Initial Ca balance including Ca compensation algorithm on 100%, Physician ordered calcium added	Designed to study effect on iPTH of keeping systemic iCa within physiological range during RCA CVVHD

*CVVH* continuous veno-venous hemofiltration, *CVVHDF* continuous veno-venous hemodiafiltration, *CVVHD* continuous veno-venous hemodialysis, *iPTH* intact parathyroid hormone

Knowledge of the effects of regional citrate anticoagulation in CRRT on vitamin D metabolism is limited. Decreased values of 25D in patients in acute renal failure were found in the van der Voort study, increasing minimally over time in both the citrate and the nadroparin groups, without statistical differences between groups [25]. In the Boer study, more than 90% of patients were vitamin D deficient at inclusion (< 50 nmol/l), and 25D decreased slightly but significantly during the study, without differences between low citrate and high citrate groups [28]. Conversely, 1,25D rose slightly during the study [28], again without differences between groups. The clinical relevance of these limited changes remains unclear.

Generally, the still limited data on the effects of citrate anticoagulation in CRRT on the endocrine system and/or bone metabolism have been reported exclusively in the early phase i.e., soon after initiating the treatment. The underlying pathophysiological data on effects on the longer term are still lacking and warrant further investigation. Systemic iCa should be targeted above 1 mmol/L to minimize the risk of increasing PTH, though there is no evidence for targeting supranormal values.

## Citrate and magnesium balance

Although the role of hypomagnesemia in critically ill patients remains open to speculation, it has been implicated as an independent risk factor for non-recovery of renal function in a cohort of critically ill AKI patients [36] and as an effector on the PTH–Ca curve [37]. Compared to heparin anticoagulation, renal replacement therapy with citrate-based regional anticoagulation increases Mg loss because, similar to calcium, magnesium is chelated by citrate and so there is increased loss in the effluent [38, 39].

Postfilter Mg supplementation is generally limited in convective modalities compared to diffusive modalities because of the higher blood flows used, necessitating higher prefilter volumes of citrate buffer to achieve therapeutic citrate concentrations [28]. More concentrated citrate buffer solution would go some way to alleviate this problem [28], while using a postfilter fluid with a higher Mg concentration (1.5 mmol/L) [40], and increasing systemic Mg supplementation based on more frequent controls also limits negative Mg flux [28, 38]. Modalities incorporating diffusive modalities, allowing lower blood flows and a lower net citrate load, such as CVVHD or CVVHDF, may therefore be advantageous when overcoming insufficient replacement of magnesium.

## Citrate and phosphate balance

Increased phosphate levels are present consistently in patients with AKI, caused by decreased renal excretion [13]. However, the adoption of CRRT has led to an increased prevalence of hypophosphatemia [41–47], due to higher clearance compared to intermittent modalities and longer circuit lifespan in citrate anticoagulation compared to heparin [3], especially when standard CRRT solutions are used. In critically ill patients, hypophosphatemia has been associated with neuromuscular disturbances, respiratory muscle dysfunction, and myocardial dysfunction [48]. Incidence of up to 80% has been reported in patients undergoing CRRT and it is particularly prevalent in high-dose CRRT (> 35 ml/kg/h) [43, 44]. During continuous hemodialysis in patients with AKI, hypophosphatemia was associated with a higher incidence of prolonged respiratory failure requiring tracheostomy, but not 28 day mortality [46]. In another retrospective study in 760 patients undergoing CVVH for AKI, hypophosphatemia was associated with increased 28 day mortality [49]. Numerous studies have demonstrated that the use of a phosphate-containing solution, in the setting of RCA, significantly reduces CRRT-related phosphate depletion [47, 50–57] though use was associated with relative hypocalcemia and metabolic acidosis in some studies [50, 58, 59]. A retrospective cohort study of patients undergoing CRRT found that use of phosphate-containing versus phosphate-free solutions was independently associated with fewer ventilator days and shorter stay in the intensive care unit [60]. It should be noted that changes in phosphate levels after initiation of CRRT can influence the interpretation of total-to-ionized calcium ratio, used as a marker for citrate accumulation [61]. More frequent checks of circulating phosphate levels should be instigated if phosphate-containing solutions are not used to reduce the incidence of hypophosphatemia and to determine the need for parenteral phosphorus supplementation. Despite only anecdotal reporting, if phosphate-containing solutions are used, care should be taken to monitor for calcium phosphate precipitation as the site of calcium reinjection in the distal part of the extracorporeal circuit.

In conclusion phosphate losses too are determined by limited replacement especially in low-dose citrate solutions. A more concentrated citrate solution that allows to deliver a higher fraction of electrolyte-rich substitution fluid might overcome the insufficient replacement of phosphate. Modalities incorporating diffusive techniques, such as CVVHD or CVVHDF, may be advantageous when overcoming insufficient replacement of phosphate.

## Citrate, inflammation, and oxidative stress

It has been documented that calcium chelation, mediated through reducing iCa, attenuates inflammation and limits oxidative stress [5]. Several studies, both in vitro and in the HD populations, describe these effects, though those are not within the scope of this review.

In the setting of CVVH, a prospective randomized study of 20 critically ill patients with AKI, studied the effects of either heparin or citrate anticoagulation on oxidative stress, measuring myeloperoxidase (MPO) (polymorphonuclear (PMN) cell degranulation and release from endothelial cells), as well as inflammatory cytokine production [62]. In the heparin group, the postfilter serum MPO levels were significantly higher than the prefilter at 6 h [62]. However, in the citrate group this increase from pre- to postfilter was absent. Citrate significantly decreased prefilter serum (thus systemic) MPO and interleukin-8 (IL-8) levels from baseline to 6 h, with significant differences between the 2 groups. Heparin provided only significant prefilter Tumor Necrosis Factor α (TNF-α) reduction with a similar trend in the citrate group and without differences between the 2 groups [62]. The authors concluded that citrate reduced both membrane bio-incompatibility-induced and systemic oxidative stress and inflammation.

Schilder et al. studied modulation of immune response in differing anticoagulation regimes (in citrate, heparin, and no anticoagulation) in CVVH. Inlet (prefilter) concentrations and mass rates of IL-6 and IL-8 decreased during CVVH, without differences and similar fluxes over the filter between groups [63]. It was concluded that the choice of anticoagulation did not increase or attenuate levels of interleukin-6 (IL-6) and IL-8 during CVVH [63]. However, the same group studied complement activation (reflected by circulating C5a levels) and neutrophil degranulation in the filter (MPO and elastase) and MPO release from endothelium in CVVH with differing anticoagulant regimes: citrate, heparin, and no anticoagulation [64]. C5a, elastase, and MPO were measured in blood samples collected pre- and postfilter and C5a was also measured in the ultrafiltrate. In the heparin group, there was more C5a production across the filter compared to other groups and net production of elastase and MPO across the filter, while production was absent in the citrate group [64]. It was concluded that citrate conferred less filter-induced, potentially harmful complement activation and neutrophil degranulation and less endothelial activation than heparin [64].

Gattas et al. studied effects of anticoagulant (citrate vs. heparin) on functional circuit life and changes in interleukin-6, interleukin-8, and interleukin-10 blood levels. In 857 circuits in 212 patients randomized between the 2 groups, regional citrate and calcium anticoagulation prolonged circuit life but demonstrated no differences in cytokine levels [65]. Table 2 provides an overview of studies describing inflammation and oxidative stress in vivo in RCA CRRT.

**Table 2:** Studies describing inflammation and oxidative stress in vivo in RCA CRRT

Author	Tiranathanagul (62)	Schilder (63)	Schilder (64)	Gattas (65)
Mode	CVVH	CVVH	CVVH	CVVH/CVVHDF
Comparison	Heparin vs citrate	Heparin vs citrate vs none	Heparin vs citrate vs none	Heparin vs citrate
Numbers included	10 vs 10 patients	8 vs 17 vs 13 patients	8 vs 17 vs 13 patients	467 vs 390 circuits in 212 patients
Markers	MPO, IL-8, TNF-α	IL-6, IL-8	C5a, elastase, MPO	IL-6, IL-8, IL-10
Outcome	At 6 h pre filter MPO in heparin group higher than baseline, not in citrate. Serum MPO significantly lower in citrate at 24 h. IL-8 at 6 h lower than baseline in citrate group, only TNF-a in heparin group, similar trend in citrate group	High inlet IL-6 and IL-8 associated with non-survival, decreased during CVVH. No difference in flux between groups	Total mass production rate of C5a elastase and MPO highest in heparin. Less filter-induced complement activation, neutrophil degranulation and endothelial activation than heparin in citrate	No differences in change from baseline to 2nd measurement between groups
Remarks	24 h study	12 h study	12 h study	Baseline and measurement at 48–72 h

*CVVH* continuous veno-venous hemofiltration, *CVVHDF* continuous veno-venous hemodiafiltration, *MPO* myeloperoxidase, *IL* interleukin, *C5a* complement fragment 5a, *TNF-α* tumor necrosis factor α

To summarize, the use of citrate in CRRT seems to render the hemofilter more biocompatible by decreasing complement activation and neutrophil degranulation in the filter. Evidence for systemic effects, particularly on inflammatory cytokine production, remains equivocal and no clinical benefits have been demonstrated in critically ill patients. Despite findings possibly highlighting a positive effect of citrate on inflammation and oxidative stress, citrate CRRT is strictly used to ensure filter patency, not as a treatment for sepsis.

## Conclusions

Citrate anticoagulation has become the preferred choice for anticoagulation in continuous renal replacement therapy. Calcium chelation by citrate as utilized in regional anticoagulation for CRRT has been proven to have systemic effects both on PTH and on calcium balance. There is at present no evidence that there is a direct effect of citrate chelation on systemic calcium metabolism manifesting as a mortality benefit or detriment. However, it should be stressed that previous guidelines recommending citrate-based CRRT were made without knowledge of the long-term metabolic effects and that until now studies describing long-term effects are absent. There is evidence that citrate plays a role in attenuating the bio-incompatibility of the dialysis membrane in CRRT. However, systemic effects, particularly on inflammatory cytokine production, remain equivocal and no clinical benefits have been demonstrated in critically ill patients. Figure 2. provides an overview of unapparent systemic effects of RCA in CRRT and recommendations to counter systemic effects of RCA in CRRT are given in Table 3.

**Table 3:** Recommendations to counter systemic effects of RCA in CRRT

Systemic effects of RCA	Intervention	Timeline	Remarks	References
Citrate accumulation/overload (T/iCa)	Patient selection, limit net citrate dose (blood flow dependent)	< 6 h depending on metabolic status	Care with patients in shock/liver failure	[4, 5, 61]
Increased iPTH	Ensure adequate systemic iCa before initiation of citrate CRRT, maintain systemic iCa > 1.0 mmol/l	Low iCa seen < 4 h after initiation		[25, 28, 32]
Negative calcium balance	Limit net citrate dose (blood flow dependent) early sampling and supplementation; calcium compensation always at 100% on CRRT machine	< 4 h	Citrate dose-dependent loss in effluent; in diffusive modalities blood flow is lower; hypercalcemia of immobilization may mask negative calcium balance in longer term	[4, 12, 25–28]
Negative magnesium balance	Limit net citrate dose (blood flow dependent), early sampling and supplementation, use of substitution fluids with higher magnesium concentration	< 12 h (dependent on initial value)	Citrate dose-dependent loss in effluent, in diffusive modalities blood flow is lower	[4, 12, 28, 38–40]
Negative phosphate balance	Early sampling and supplementation, use of substitution fluids with higher phosphate concentration	< 12 h (dependent on initial value)	Monitor for possible calcium phosphate precipitation at the site of calcium reinjection point when using higher phosphate-containing fluids	[47, 50–60]

Take-home messages.

- To prevent a negative calcium balance and increase in PTH: target a systemic iCa > 1,0 mmol/l
- To limit calcium loss in effluent: limit citrate dose by limiting the blood flow
- To minimize the effects of predilution: preferably use solutions with higher citrate doses
- Utilize postfilter/dialysate solutions with adequate amounts of phosphate and magnesium to counter losses via the effluent.
- Diffusive modalities generally utilize lower blood flows, contributing to a lower net citrate dose.

## Data Availability

Not applicable.

## Abbreviations

AKI: Acute kidney injury
Ca: Calcium
CRRT: Continuous renal replacement therapy
CVVH: Continuous veno-venous hemofiltration
CVVHD: Continuous veno-venous hemodialysis
CVVHDF: Continuous veno-venous hemodiafiltration
IL: Interleukin
iCa: Ionized calcium
iPTH: Intact PTH
Mg: Magnesium
MPO: Myeloperoxidase
noxPTH: Non-oxidized PTH
oxPTH: Oxidized PTH
pfiCa: Postfilter ionized calcium
PTH: Parathyroid hormone
RCA: Regional citrate anticoagulation
TNF-α: Tumor necrosis factor α
1,25D: 1,25-Dihydroxy vitamin D
25D: 25-Hydroxy vitamin D
